# Nonlinear Fatigue Damage Model of Asphalt Mixture Based on Dynamic Modulus and Residual Strength Decay

**DOI:** 10.3390/ma12142236

**Published:** 2019-07-11

**Authors:** Hongfu Liu, Xinyu Yang, Chengdong Xia, Jianlong Zheng, Tuo Huang, Songtao Lv

**Affiliations:** 1 Key Laboratory of Road Structure and Material of Ministry of Transport (Changsha), Changsha University of Science & Technology, Changsha 410114, China; 2 College of Traffic and Transportation Engineering, Changsha University of Science & Technology, Changsha 410114, China

**Keywords:** asphalt mixture, fatigue damage model, residual strength, dynamic modulus, damage evolution

## Abstract

In order to describe the fatigue damage state of asphalt mixture more reasonably, direct tensile tests of the fatigue and the residual strength under stress levels of 1.00 MPa, 0.50 MPa and 0.25 MPa with five parallel tests were carried out. The trabecular specimens of AC-13C asphalt mixture (25 cm × 5 cm × 5 cm) were manufactured with Styrene-Butadiene-Styrene (SBS) modified asphalt, aggregate basalt and limestone mineral filler. The optimum asphalt-aggregate ratio was 5.2%. The dynamic modulus decay and the residual strength decay were termed as the damage variables to evaluate the fatigue damage process of asphalt mixtures, respectively. Based on the test results, the decay patterns of the dynamic modulus and the residual strength during fatigue tests under different stress states were revealed, and the model and the parameters of fatigue damage according to the corresponding decay patterns were obtained. Then, based on the assumption that the residual strength and dynamic modulus depend on the same damage state, the relationship between the two damage definitions was given, and the residual strength-dynamic modulus coupled model was established. The results showed that the residual strength-dynamic modulus coupled model could better describe the fatigue damage evolution law of asphalt mixture, and the parameter of this coupled model could be obtained by less residual strength tests. A modified formula for calculating the damage variables associated with residual strength and dynamic modulus was proposed based on the relationship between two kinds of damage variables.

## 1. Introduction

Fatigue damage of asphalt pavements is a complex phenomenon, which occurs as a result of repeated traffic loading [1,2,3]. In asphalt pavements, the vehicular cyclic loads create areas of tensile stresses at the bottom of the pavement, which is contributed to the formation of microcracks. Damage initiates with the formation of microcracks. These microcracks are coalesced and developed into larger visible cracks under repeated traffic loads. Then the damage intensifies and cracks propagate upward to the surface of the pavement in the form of alligator cracking.

Quantitative assessment of fatigue damage is a critical issue that needs to be addressed both in the mixtures and pavement designs [4]. Many different approaches were mentioned in the literature to quantify the fatigue damage process of the asphalt mixture. Saboo et al. [5] developed a new phenomenological model, which could accurately predict the fatigue life of mixtures by studying the existing phenomenological models. María Castro et al. [6] combined continuous damage theory with the phenomenological method to establish a model based on continuous damage theory, which could estimate standard fatigue curves with fewer data. Shen S. et al. [7] and Bhasin et al. [8] used the energy dissipation method to evaluate the fatigue cracking of asphalt mixture and found that the energy method could accurately predict the cracking life of different materials. Liu G. et al. [9] used the 4-point bending beam fatigue test under energy control mode to evaluate the fatigue damage characteristics of different content of recycled asphalt mixture. Lv S. et al. [10] established a unified description model of fatigue equation of asphalt mixture under low temperature and low loading frequency, which means that the fatigue equations and curves under different loading frequencies could be characterized by one equation and one curve. Sadek et al. [11] established a probabilistic framework that included the viscoelastic continuous damage method and considered the variability of input parameters. The analysis results under this framework were more consistent and reliable than those under deterministic viscoelastic continuous damage analysis. Babadopulos et al. [12] coupled the aging model with the simplified viscoelastic continuous damage model based on the work potential theory and successfully incorporated aging into the mixture fatigue damage model. Cao W. et al. [13] used the viscoelastic continuous damage method to analyze the results of LAS and TS tests. It was found that the relationship between damage characteristics and test methods and strain distribution was slightly dependent, and the degree of dependence was material specific. Mello, L.G.R. d et al. [14] applied the continuous damage theory to the bending fatigue test to evaluate its applicability and proposed a practical method for predicting fatigue behavior by comparing the effects of different parameters and coefficients obtained in the research on the fatigue behavior of the asphalt mixture. Moreno-Navarro F. et al. [15] proposed a new approach which combines the study of the changes produced in the geometry and the energy dissipated by the material in each load cycle and provided a more refined analysis of fatigue damage in asphalt mixtures. Among different approaches, phenomenological approaches, which associated stress or strain at the bottom of the asphalt layer with repeated loading times leading to fatigue, were widely used in structural design and fatigue performance analysis of conventional asphalt pavement because of their simplicity. Unfortunately, this method could hardly describe the damage process and structure degradation of asphalt materials. Fracture mechanics methods were used to study fatigue by monitoring the crack length development and propagation followed by fracture failure. However, the initiation and the propagation of microcracks or damage occupied most of the fatigue life, so it was critical to apply the damage approach to explain the fatigue damage evolution process of asphalt mixtures. When using the damage analysis method, the first and the most basic problem was to define an appropriate damage variable to describe the damage state of materials. The fatigue development process and critical damage value described by different damage variables were also different [16]. Shen S. and Carpenter et al. [17,18] utilized the ratio of dissipated energy change (RDEC) as an indicator for assessing the resistance to fatigue damage of asphalt mixtures. Kim et al. [19] used the viscoelastic continuous damage model to analyze the fatigue damage characteristics of asphalt mixtures. Jiang et al. [20] and Ni et al. [21] applied the damage variable defined by residual strength decay to the study of fatigue damage characteristics in asphalt mixtures. The fatigue damage model could reflect the damage state of asphalt mixtures more accurately. However, the definition of damage based on the dissipated energy method and continuous damage model method is too complex to be obtained directly from the test, and the measurement of residual strength is not continuous, therefore time- consuming. Considering that strength degradation is the most direct reason for structure failure, taking the residual strength as a fatigue damage variable is worth studied.

During the fatigue test, the dynamic modulus decreases with the continuous evolution and accumulation of internal damage, and the dynamic modulus decay is a damage variable that can be continuously measured. Therefore, the dynamic modulus is also a universal variable to describe fatigue damage of asphalt mixture. However, the existing models of residual strength and dynamic modulus are often proposed independently, and most of them lack a good consideration about the relationship between them, which results in a large number of tests when studying the fatigue behavior of asphalt mixture and describing its fatigue damage state. In addition, the residual strength test is destructive, and only one residual strength value can be obtained for each specimen. The test cost is very high. Moreover, there are individual differences among the specimens, and the comparability between the residual strength of different specimens is poor. Consequently, it is necessary to study the relationship between the two damage variables and propose a method for defining the damage variable with good applicability and high reliability.

**Figure 1:**
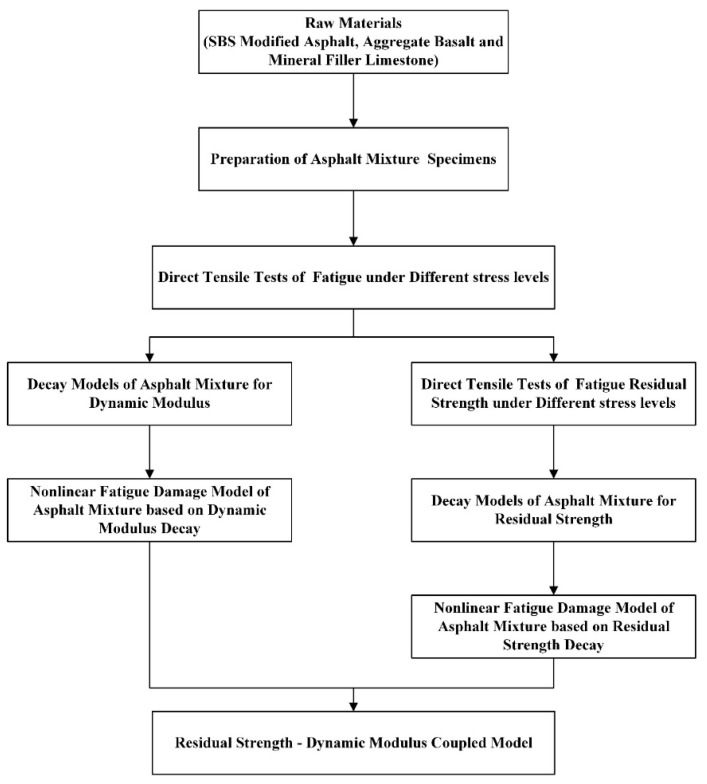
Flowchart of the work.

Different test conditions mean different stress states [22]. For complex stress states, the internal structure is affected by factors such as stress redistribution, residual stress, and difficulty in accurately capturing stress (strain). These factors therefore make the internal structure challenging to analyze. Compared with other fatigue test methods, the stress state of the specimen in direct tension test is relatively single. Moreover, the internal stress-strain relationship is more natural to analyze. In the United States, in recent years, new fatigue test methods have been developed. Viscoelastic continuum damage theory was applied to simulate the asphalt behavior in direct tension [23]. The corresponding test method standard is AASHTO TP107. Behrooz K. et al. [24] presented a new method for simulating the behavior of asphalt concrete also in uniaxial tension. Besides, Lv S. et al. [25] simulated modulus decay modes under different loading conditions by using a non-linear fatigue damage model, which indicated that tensile failure was the main reason of fatigue damage for asphalt mixture. Therefore, in this paper, the analysis was conducted according to the flowchart, as shown in Figure 1. This research presents direct tensile fatigue test and fatigue residual test of asphalt mixtures at different stress levels. The non-linear fatigue damage models of asphalt mixtures with dynamic modulus decay and residual strength decay as damage variables were established based on the dynamic modulus decay model and residual strength decay model, respectively. Under the assumption that the residual strength and dynamic modulus at the same time depend on the same damage state inside the material, the coupled relationship between the residual strength and dynamic modulus was given, and the coupled model of residual strength and dynamic modulus was established. The paper proposes a modified formula for calculating the damage variables associated with residual strength and dynamic modulus based on the relationship between two kinds of damage variables.

## 2. Materials and Specimens Preparation

In this paper, the strength and fatigue tests of the direct tensile were carried out. The asphalt mixture AC-13C which composed of Styrene-Butadiene-Styrene (SBS) modified asphalt, aggregate basalt and limestone mineral filler was selected. The performance indexes of SBS modified asphalt, the properties of aggregate basalt and the properties of mineral filler limestone are shown in Table 1, Table 2 and Table 3, respectively. The test results, show that SBS modified asphalt and the aggregate satisfied the requirements of Technical Specifications for Construction of Highway Asphalt Pavement (JTG F40-2004) [26], which is the technical specifications for asphalt pavement construction in China.

**Figure 2:**
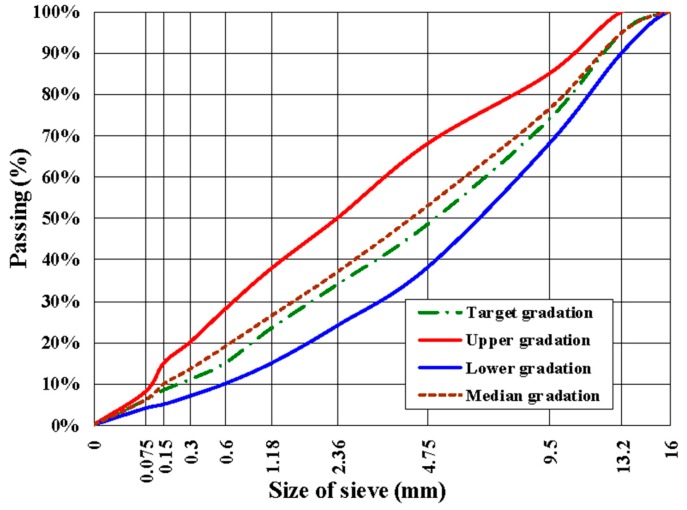
Aggregate gradation curve of dense graded asphalt mixture (AC-13C).

According to the Specifications for Design of Highway Asphalt Pavement (JTG D50-2017) [27], dense skeleton aggregate gradation was selected. Detailed information is shown in Table 4 and Figure 2, respectively. The optimum asphalt-aggregate ratio was 5.2%, which was obtained by the Marshall Tests. The test results are shown in Table 5. According to the Specifications and Test Methods of Asphalt and Asphalts Mixtures for Highway Engineering (JTG E20-2011) [28], the rutting plate specimens with length, width, and height of 30 cm × 30 cm × 5 cm were compacted by the asphalt mixing wheel and cut into 25 cm × 5 cm × 5 cm trabecular specimens. There were five parallel tests for each type of test.

## 3. Test Method

### 3.1. Direct Tensile Fatigue Test

**Figure 3:**
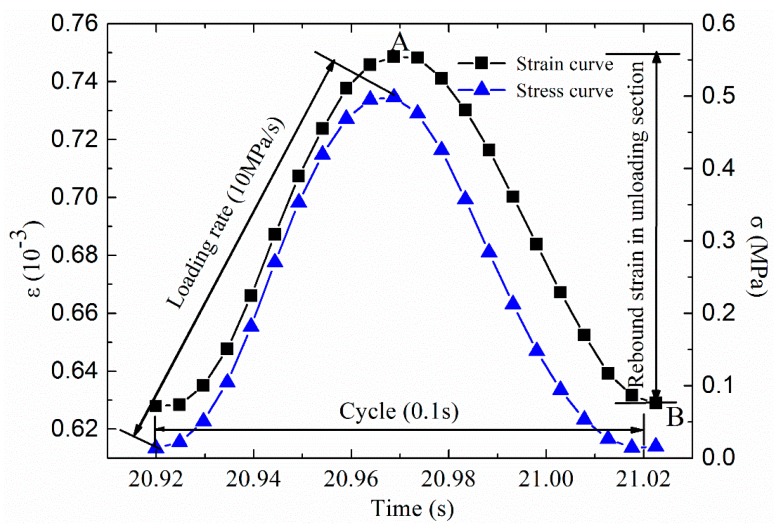
The curves of stress and strain with time.

The material testing system (MTS) was employed in the tests. The maximum tension and compression loads are 100 kN, and the average error in the range is less than 0.5%. Each stress cycle of the force and displacement values are automatically collected by the data acquisition system. The corresponding stress and strain calculation formulas are shown in Equations (1) and (2), and the curves of stress and strain with time in a period are shown in Figure 3. The time interval of the data acquisition system can be set according to the requirements of the experimenter. In this paper, the time interval of the data acquisition system is 200 Hz. A total of 15 trabecular specimens were tested in direct tensile fatigue test.

(1) $$\sigma =\frac{F}{A}$$

(2) $$\varepsilon_{ei}=\frac{U_{\max\;i}-U_{\min\;i}}{L}$$

where *σ* is the stress; *F* is applied load; *A* is the radial cross-sectional area of the specimen in a non-destructive state; *ε_ei_* is the rebound strain per cycle, as shown in Figure 3 in the strain value of the AB section; *U*_max *i*_ is the peak displacement; *U*_min *i*_ is the valley displacement; *L* is the length of the test specimen, and *L* is 25 cm in this paper.

**Figure 4:**
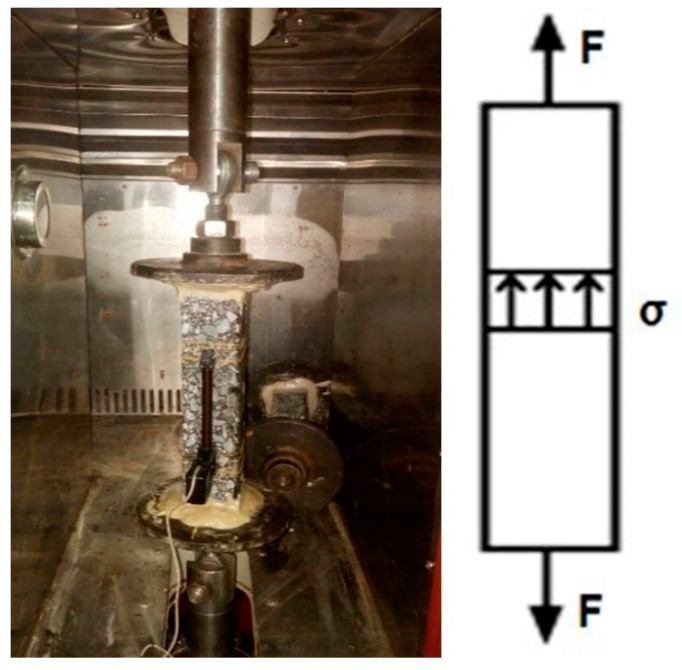
Direct tensile test and force diagram.

The design scheme and main control criteria of direct tension fatigue test are as follows:

(1) Loading mode: stress control mode, the direct tensile stress level is 1.00 MPa, 0.50 MPa, 0.25 MPa;
(2) Loading waveforms and frequencies: without considering the 10 Hz continuous alternating sinusoidal waveforms of load intermission time, the loading waveforms with stress levels of 0.5 MPa are shown in Figure 3.
(3) Test temperature: 15 ± 1 °C, the specimen was kept for more than 12 h at this temperature before the test. The direct tensile fatigue test of trabecular specimens using material test system is presented in Figure 4.

### 3.2. Fatigue Residual Strength Test

The fatigue residual strength test needs to determine the initial strength, fatigue life, and residual strength after different fatigue times. In order to determine the different fatigue times in the residual strength test, two principles are generally followed. Firstly, the fatigue times should not be too large, which often leads to fatigue failure of the specimens without reaching the predetermined number of times, and greatly reduces the success rate of the test. Secondly, there should be a certain gap in determining the fatigue times of different residual strength, so that the test results can fully reflect the decay law of residual strength. The selection of the standard value of fatigue life in residual strength test is one of the key factors affecting the test results. The standard value of fatigue life used in this study is the average value of fatigue life obtained from direct tensile fatigue test. The average fatigue life under repeated loading is 20%, 50%, 65%, and 80% of the fatigue times. After unloading for 3 s, the dynamic load strength of the specimens was measured at the loading rate consistent with the fatigue test, that was, the residual strength results of different fatigue times.

Direct tension fatigue was used in the fatigue residual strength test. The loading frequency is 10 Hz. The test temperature is 15 °C. The test procedures are as follows: (1) The initial dynamic load strength was tested under three different stress levels 1 MPa, 0.5 MPa and 0.25 MPa, and the loading rates under different stress levels were 20 MPa/s, 10 MPa/s, and 5 MPa/s, respectively. (2) The residual strength of the specimen after repeated fatigue was measured, and the loading rate of the residual strength was consistent with that of the initial strength. A total of 75 trabecular specimens were tested in fatigue residual strength test.

## 4. Test Result and Analysis

The direct tensile fatigue test results of three different stress levels of 1 MPa, 0.5 MPa, and 0.25 MPa are summarized in Table 6.

### 4.1. The Decay Model for Dynamic Modulus

The dynamic modulus is the ratio of the axial stress to the recoverable axial rebound strain. The dynamic modulus could be calculated as follows [29]:

(3) $$E=\frac{\sigma }{\varepsilon_{ei}}$$

where *E* is the dynamic modulus; *σ* is the stress level imposed by fatigue test; *ε_ei_* is the rebound strain per cycle.

Fatigue test data are generally large. A program for fatigue test data analysis is developed by MATLAB software. The peak, valley and corresponding time of load and displacement in each cycle are extracted conveniently and quickly, and then the dynamic modulus of each cycle is calculated.

#### 4.1.1. The Initial Values of Dynamic Modulus at Different Fatigue Stress Levels

The moduli of the 50th cycle are widely used as the initial moduli of fatigue test [30]. However, the fatigue cycles of the same material vary at different stress levels, so the initial moduli values obtained by this method have a large deviation. In this paper, the initial dynamic modulus *E*_0_ is defined as the average one of the five dynamic moduli, which was near the cycle ratio *N*/*N_f_* = 0.01. The average initial dynamic modulus values of five parallel specimens under different stress levels are summarized in Table 7.

Table 7 shows that the coefficient of variation and the initial value of dynamic modulus obtained by parallel fatigue test at the same stress level. As can be seen, the values of the coefficient of variation are small, and the initial value of dynamic modulus increase slightly with the increase of stress level.

#### 4.1.2. The Critical Value of Dynamic Modulus at Different Fatigue Stress Levels

During the fatigue tests, the modulus decreased with the increase of the load cycles until the failure of the specimens. The critical value refers to the damage value when the specimen gets fatigue failure at the end of the fatigue test. In this paper, the average value of the dynamic modulus in the last five loading cycles in the fatigue tests were taken as the critical dynamic modulus value *E*_min_. The average critical dynamic modulus values of five parallel specimens under different stress levels are summarized in Table 8.

Table 8 shows that the critical value of dynamic modulus *E*_min_ increases with the increase of stress level. The fatigue failure of the specimen has a larger modulus in the fatigue test with a higher stress level.

#### 4.1.3. Establishment of the Decay Model for Dynamic Modulus 

**Figure 5:**
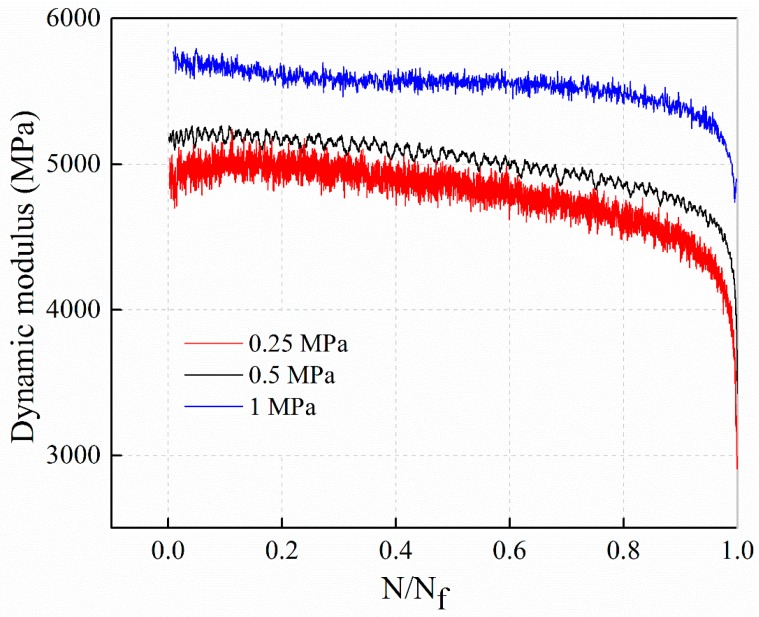
Decay law of dynamic modulus at different stress levels.

When a stress controlled fatigue tested, the initial decrease of the modulus is mainly related to the effect of permanent deformations, which can neutralize the real damage produced by the fatigue process [31]. Therefore, in this paper, the decay law of dynamic modulus at different stress levels is shown in Figure 5. From top to bottom, the dynamic modulus decay curves of stress level 1 MPa, 0.5 MPa, and 0.25 MPa are obtained.

Figure 5 illustrates, the dynamic modulus decay law for AC-13C mixtures under different stress levels can be roughly divided into two stage. In the first stage, the dynamic modulus decay rate is stable, and in the second stage, when the cycle ratio is about 90%, the decay rate accelerates and enters a sharp decay stage. Also, the attenuation of dynamic modulus in the fatigue process do not satisfy the linear law and has definite non-linear characteristics. In order to facilitate comparison, the dynamic modulus decay and cyclic ratio are normalized, and it is assumed that the dynamic modulus decay and cyclic ratio satisfies the following power function relations, as shown in Equation (4).

(4) $$\frac{E\left(N\right)-E_{\min}}{E_{0}-E_{\min}}={\left(1-\frac{N}{N_{f}}\right)}^{m}$$

where *E*(*N*) is dynamic modulus; *E*_0_ is the initial value of dynamic modulus; *E*_min_ is the critical value of dynamic modulus; *N* is the number of fatigue load cycles; *N_f_* is the fatigue life; *m* is the power exponent of modulus decay law, 0 < *m* < 1.

The initial value of dynamic modulus *E*_0_ represents the initial fatigue state of asphalt mixture, that is, when *N* = 0, there is *E*(*0*) = *E*_0_; the critical value of dynamic modulus *E*_min_ represents the fatigue failure state of asphalt mixture, that is, when *N* = *N_f_*, there is *E*(*N_f_*) = *E*_min_. Equation (4) could also be expressed as Equation (5) shows.

(5) $$E\left(N\right)=\left(E_{0}-E_{\min}\right){\left(1-\frac{N}{N_{f}}\right)}^{m}+E_{\min}$$

When establishing the dynamic modulus decay model, the boundary condition of the dynamic modulus, and the regularity of the decreasing dynamic modulus should be satisfied. The boundary conditions and decay patterns of the dynamic modulus decay model are verified, as shown in Equation (6).

(6) $$\left\{ \begin{array}{l} E\left(0\right)=E_{0}\\ E\left(N_{f}\right)=E_{\min}\\ \frac{dE\left(N\right)}{dN}=\left(E_{0}-E_{\min}\right)m{\left(1-\frac{N}{N_{f}}\right)}^{m-1}\left(-\frac{1}{N_{f}}\right)<0\\ \frac{d^{2}E\left(N\right)}{dN^{2}}=\left(E_{0}-E_{\min}\right)\frac{m}{N_{f}}\left(m-1\right){\left(1-\frac{N}{N_{f}}\right)}^{m-2}\left(-\frac{1}{N_{f}}\right)<0 \end{array}\right.$$

Equation (6) shows that the dynamic modulus decay model Equation (5) satisfies the boundary conditions and decreases with the increase of fatigue number. The second derivative is less than zero, which shows that the model reflects the non-linear characteristics of dynamic modulus decay.

**Figure 6:**
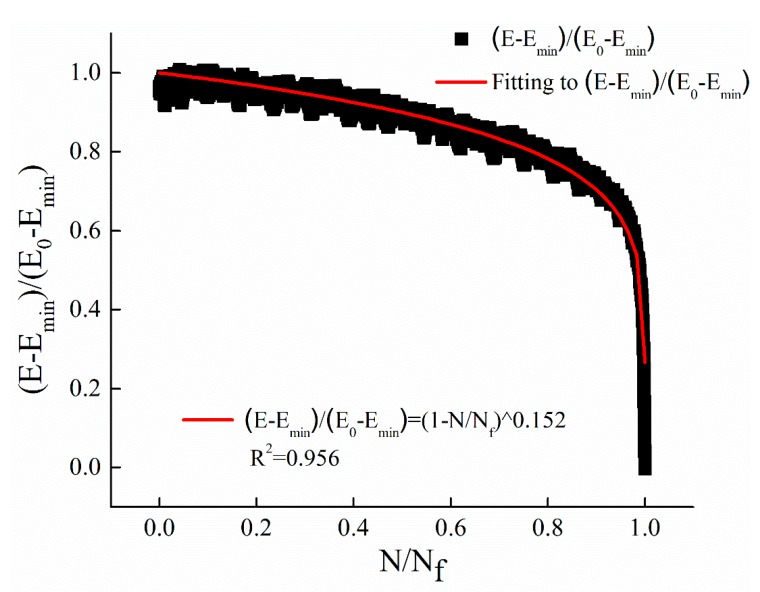
The regression results with stress levels of 0.25 MPa.

**Figure 7:**
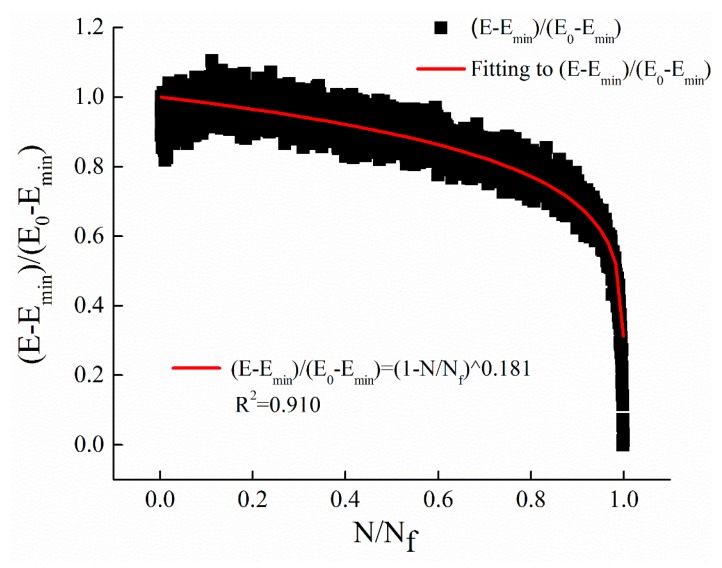
The regression results with stress levels of 0.5 MPa.

**Figure 8:**
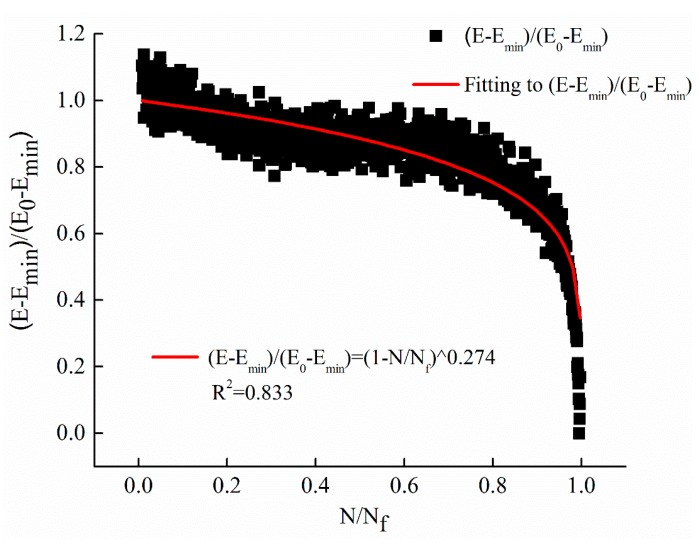
The regression results with stress levels of 1 MPa.

Equation (5) is selected to fit the decay curves of dynamic modulus with the cyclic ratio at different stress levels. The fitting results of stress levels of 0.25 MPa, 0.5 MPa, and 1 MPa are illustrated in Figure 6, Figure 7 and Figure 8, respectively. The parameters *m* of the power function decay model of the fitted dynamic modulus are summarized in Table 9.

**Figure 9:**
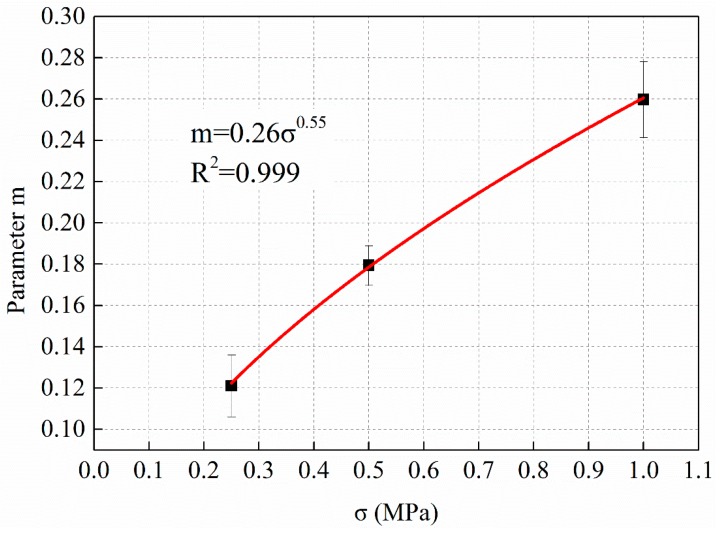
The relationship between dynamic modulus decay parameter m and stress level σ.

From Figure 6, Figure 7 and Figure 8 and Table 9, it can be observed that the power function decay model fits well with the experimental data, and the correlation coefficient R^2^ is higher. The dynamic modulus decay parameter *m* increases with the increase of stress level, and the dynamic modulus decays faster with the increase of stress level. The relationship between *m* value and stress level σ are shown in Figure 9.

Figure 9 shows that the dynamic modulus decay parameter *m* increased with the increase of stress level σ. By fitting the two parameters with power function growth law, the regression equation is shown in Equation (7).

*m* = 0.26σ^0.55^, R^2^ = 0.999 (7)

### 4.2. The Decay Model for Fatigue Residual Strength

The standard value of fatigue life in fatigue residual strength test is the average value of fatigue life obtained from direct tensile fatigue test. The number of design fatigue under different stress levels are shown in Table 10. The loading rate of residual strength after different design fatigue times is consistent with the loading rate of fatigue test. The measured residual fatigue strength is the dynamic load strength considering the speed characteristics of the asphalt mixture strength.

Similar to the dynamic modulus decay model, a power function model, as Equation (8) shows, was selected to fit the residual strength decay law.

(8) $$\frac{S_{r}-S_{rc}}{S_{0}-S_{rc}}=\frac{S_{r}-\sigma_{\max}}{S_{0}-\sigma_{\max}}={\left(1-\frac{N}{N_{f}}\right)}^{u},\;0<u<1$$

where *S_rc_* is the critical residual strength at failure, it is generally believed that failure occurs when the residual strength *S_rc_* equals the applied load *σ*_max_, that is, *S_rc_* = *σ*_max_; *u* is the undetermined parameter of model fitting; *S_r_* and *S*_0_ are residual strength and initial strength respectively; *σ*_max_ is the maximum stress of fatigue load.

When *N* = 0, it represents the initial state of the material, *S_r_*(0) = *S*_0_, *S*_0_ is the initial value of dynamic load strength of asphalt mixture; when *N* = *N_f_*, then *S_r_*(*N_f_*) = *σ*_max_, *σ*_max_ represents the residual strength of fatigue failure. Equation (8) could also be expressed as Equation (9) shows.

(9) $$S_{r}(N)=(S_{0}-\sigma_{\max}){\left(1-\frac{N}{N_{f}}\right)}^{u}+\sigma_{\max}$$

When establishing the dynamic modulus decay model, the boundary conditions and decay laws of the decay model have been verified. The model can satisfy the boundary conditions and reflect the nonlinear characteristics of decay. The model is characterized by the primary conditions of the residual strength model, and the model parameters are simple and easy to determine.

**Figure 10:**
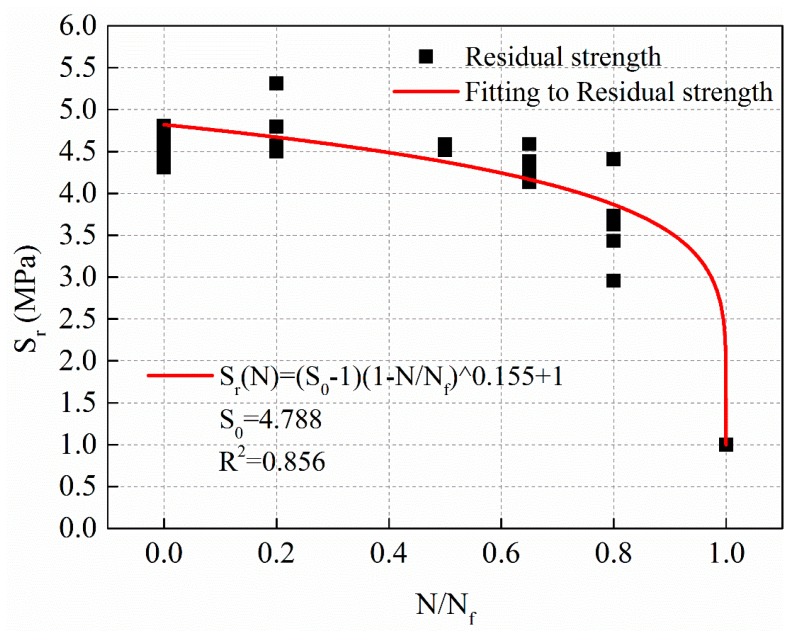
The decay law of residual strength with stress levels of 1 MPa.

The relationship between the residual strength and the cycle ratio is fitted by the Equation (9). The fitting curve of stress levels of 1 MPa is shown in Figure 10, and the regression result of residual strength with a power function of cycle ratio is shown in Equation (10).

(10) $$S_{r}(N)=\left(S_{0}-1\right){\left(1-\frac{N}{N_{f}}\right)}^{0.155}+1,S_{0}=4.788,R^{2}=0.856$$

**Figure 11:**
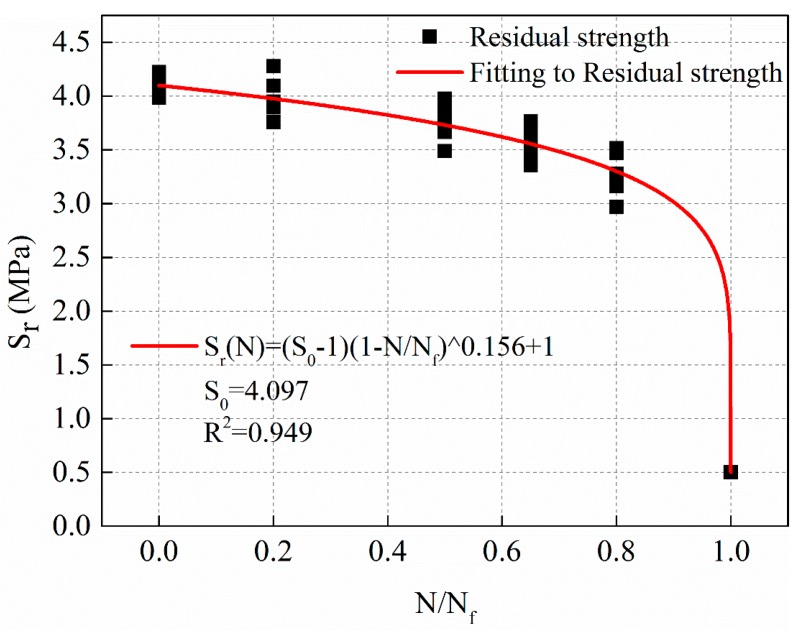
The decay law of residual strength with stress levels of 0.5 MPa.

The fitting curve of stress levels of 0.5 MPa is shown in Figure 11, and the regression result of residual strength with a power function of cycle ratio is shown in Equation (11).

(11) $$S_{r}(N)=\left(S_{0}-0.5\right){\left(1-\frac{N}{N_{f}}\right)}^{0.156}+0.5,S_{0}=4.097,R^{2}=0.949$$

**Figure 12:**
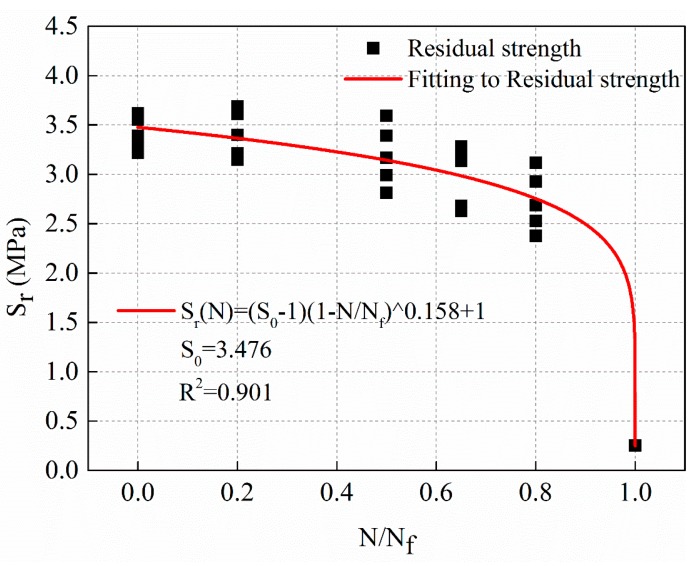
The decay law of residual strength with stress levels of 0.25 MPa.

The fitting curve of stress levels of 0.25 MPa is shown in Figure 12, and the regression result of residual strength with a power function of cycle ratio is shown in Equation (12).

(12) $$S_{r}(N)=\left(S_{0}-0.25\right){\left(1-\frac{N}{N_{f}}\right)}^{0.158}+0.25,S_{0}=3.476,R^{2}=0.901$$

The initial strength $S_{0}$ in Equations (10)–(12) was obtained by curve fitting.

Figure 10, Figure 11 and Figure 12 show that the power function decay model of residual strength can better reflect the slow decay of the material at the initial stage of fatigue. The strength of the material decreases rapidly until the final critical break.

### 4.3. Fatigue Damage Model

#### 4.3.1. Establishment of the Fatigue Damage Model Based on Dynamic Modulus Decay Law

Since there are different critical damages when a failure occurs under different conditions, it is meaningful to study the law of damage threshold and its influencing factors to accurately predict failure. The critical damage variable defined by the dynamic modulus could be calculated as Equation (13) shows.

(13) $$D_{cf}(N)=1-\frac{E_{\min}}{E_{0}}$$

where *D_cf_* is the critical damage variable; *E*_min_ is the critical value of dynamic modulus; *E*_0_ is the initial value of dynamic modulus.

**Figure 13:**
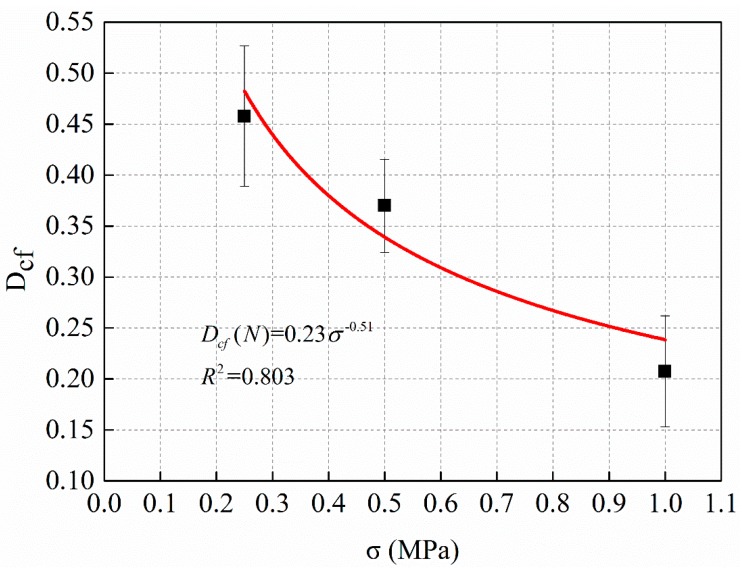
The relationship between critical damage variable and stress level σ.

From the data of Table 7 and Table 8, the critical damage variable defined by the dynamic modulus is calculated according to the Equation (13), and the relationship between critical damage variable and stress level *σ* is shown in Figure 13.

Figure 13 shows that the relationship between the critical damage variable calculated by the definition of dynamic modulus and the stress level *σ* satisfies the power function relationship. The power function regression equation is shown in Equation (14).

(14) $$D_{cf}=0.23\sigma^{-0.51}$$

By using the effective stress equivalence principle of the constitutive relation, that is, the Lemaitre strain equivalence principle [32]. The damage variables can be used for the dynamic modulus and the initial dynamic modulus of the unloading section of each cycle in the fatigue process, as shown in Equation (15).

(15) $$D_{f}(N)=1-\frac{E(N)}{E_{0}}$$

where *D_f_*(*N*) is the damage variable defined by the decay of the dynamic modulus; *E*(*N*) is the dynamic modulus of the unloading section per cycle during fatigue progress; *E*_0_ is the initial value of dynamic modulus.

Based on the definition of damage variation and dynamic modulus decay law, the fatigue damage model could be built as Equation (16) shows.

(16) $$\begin{array}{l} D_{f}(N)=1-\frac{E(N)}{E_{0}}=1-\frac{\left(E_{0}-E_{\min}\right){\left(1-\frac{N}{N_{f}}\right)}^{m}+E_{\min}}{E_{0}}\\ =\left(1-\frac{E_{\min}}{E_{0}}\right)\left[1-{\left(1-\frac{N}{N_{f}}\right)}^{m}\right]\\ =D_{cf}\left[1-{\left(1-\frac{N}{N_{f}}\right)}^{m}\right] \end{array}$$

where *D_f_*(*N*) is the damage variable defined by the decay of the dynamic modulus; *D_cf_* is the critical damage variable; *N* is the fatigue cycle number; *N_f_* is the Fatigue life; *m* is the fatigue damage model fitting parameter, which is consistent with the parameter *m* of the dynamic modulus power function decay model.

In order to understand the change rule of fatigue damage more clearly, Equation (16) is transformed, as shown in Equation (17).

(17) $$\frac{D_{f}(N)}{D_{cf}}=1-{\left(1-\frac{N}{N_{f}}\right)}^{m}$$

**Figure 14:**
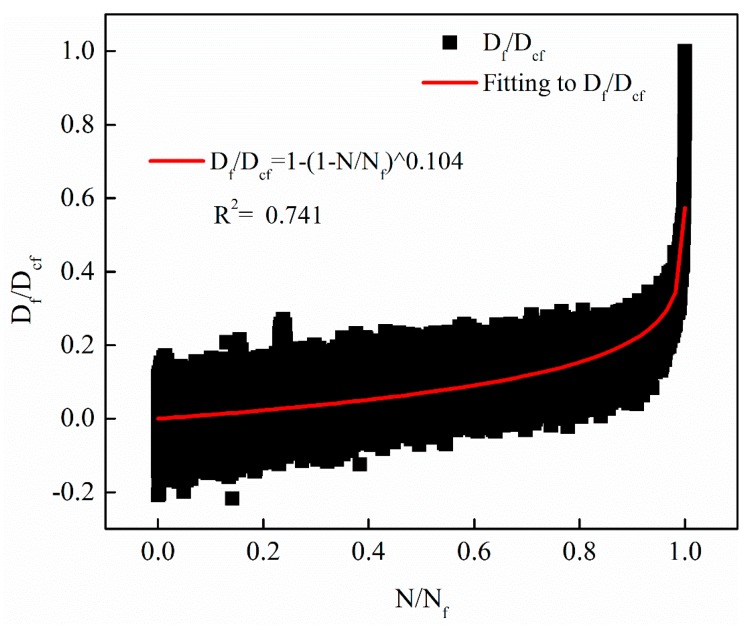
The evolution curves with stress levels of 0.25 MPa.

**Figure 15:**
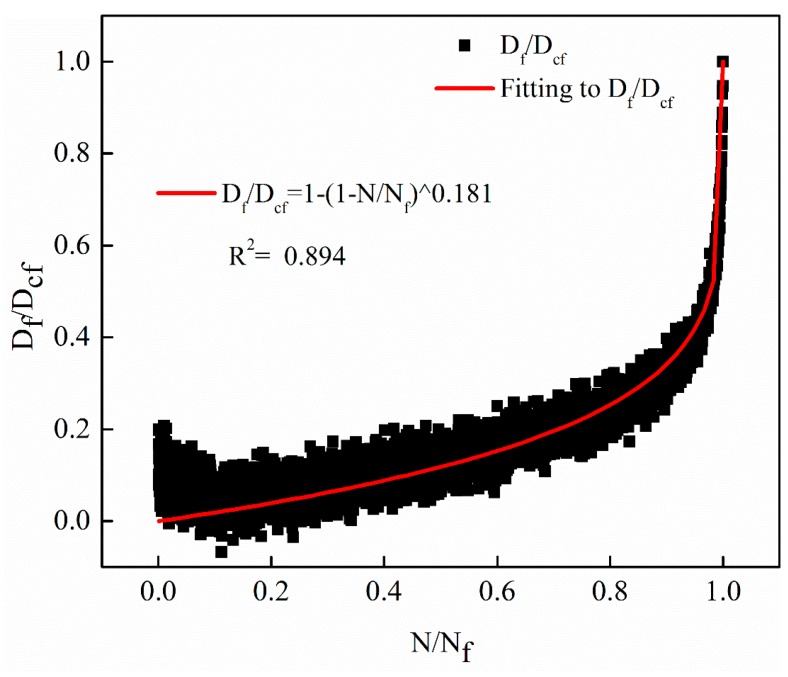
The evolution curves with stress levels of 0.5 MPa.

**Figure 16:**
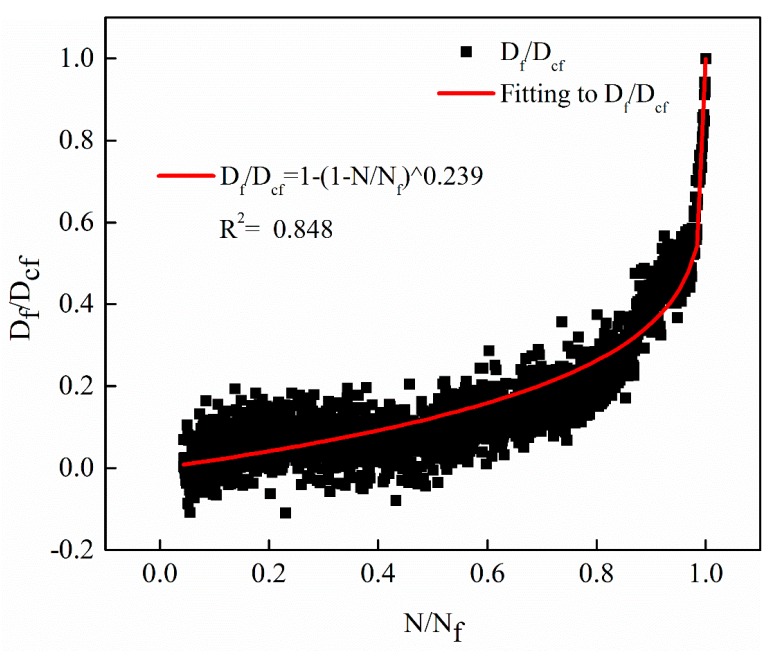
The evolution curves with stress levels of 1 MPa.

The evolution curves of the fatigue damage rate *D_f_*(*N*)/*D_cf_* based on the decay of the dynamic modulus with different stress levels were verified by the Equation (17). The verification results of the three different stress levels of 0.25 MPa, 0.5 MPa and 1 MPa are shown in Figure 14, Figure 15 and Figure 16, respectively. Note that the model parameter *m* is consistent with the parameters determined when fitting the decay law of the dynamic modulus.

From Figure 14, Figure 15 and Figure 16, it can be observed that the proposed non-linear fatigue damage model is in good agreement with the original fatigue test data. The fatigue damage has prominent non-linear characteristics with the change of cycle ratio, which reveals the evolution law of non-linear fatigue damage of asphalt mixture.

By substituting Equations (7) and (14) into Equation (16), the nonlinear fatigue damage evolution Equation (18) is obtained.

(18) $$\begin{array}{l} D_{f}(N)=D_{cf}\left[1-{\left(1-\frac{N}{N_{f}}\right)}^{m}\right]\\ =\left(0.23\sigma^{-0.51}\right)\left[1-{\left(1-\frac{N}{N_{f}}\right)}^{0.26\sigma^{0.55}}\right] \end{array}$$

**Figure 17:**
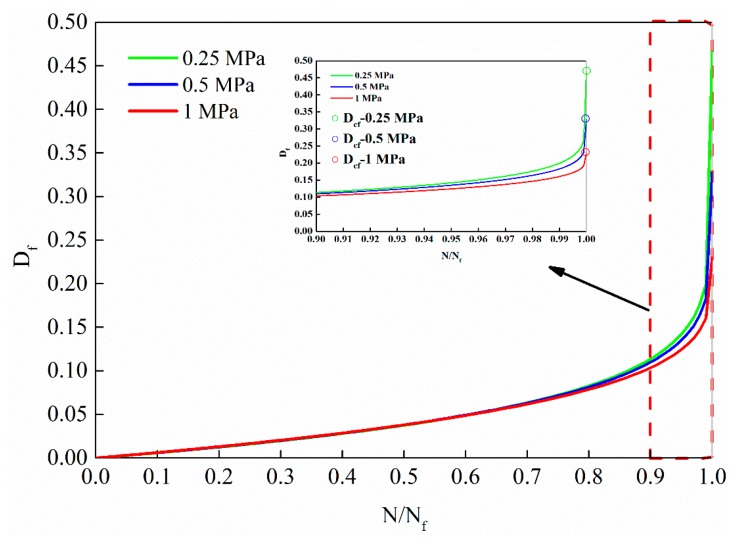
The damage evolution law based on dynamic modulus decay under different stress levels.

The damage evolution law under different stress levels is shown in Figure 17.

It can be observed from Figure 17 that the fatigue damage evolution law under different stress levels is that at the beginning of the cycle ratio, the damage increases slowly. With the increase of the cycle ratio, the rate of damage increases gradually, especially in the later stage of the cycle ratio, the growth rate increases sharply. The fatigue damage evolution curves from bottom to top are 1 MPa, 0.5 MPa and 0.25 MPa, respectively. The higher the stress level, the lower the damage evolution curve.

#### 4.3.2. Establishment of the Fatigue Damage Model Based on Residual Strength Decay Law

The residual strength test after different fatigue times show that the strength gradually decreases with the increase of the fatigue frequency, which is consistent with the performance degradation law under the repeated load during the fatigue process. Therefore, it is appropriate to select the index of residual strength. The residual strength is the index of macroscopic physical and mechanical properties, to describe the degree of damage. It can macroscopically characterize the changes in the internal structure of the asphalt mixture damage. As Equation (19) describes, the damage variable *D_s_*(*N*) is termed as the ratio of strength decrement (*S*_0_ − *S_r_*(*N*)) and initial tensile strength *S*_0_.

(19) $$D_{s}(N)=1-\frac{S_{0}-S_{r}(N)}{S_{0}}=1-\frac{S_{r}(N)}{S_{0}}$$

where *D_s_(N)* is a damage variable based on residual strength decay.

It is assumed that the initial damage of the specimen is 0, The initial strength *S*_0_ is obtained by fitting the decay law of residual strength. The initial strength *S*_0_ of 1 MPa, 0.5 MPa, and 0.25 MPa are 4.788 MPa, 4.097 MPa, and 3.476 MPa, respectively. Fatigue occurs when the residual strength *S_r_*(*N*) is reduced to the stress level *σ*_max_.

Based on the definition of damage variation and residual strength decay law, the fatigue damage model could be built as Equation (20) shows.

(20) $$\begin{array}{l} D_{s}\left(N\right)=1-\frac{S_{r}}{S_{0}}=1-\frac{(S_{0}-\sigma_{\max})(1-N/N_{f}{)}^{u}+\sigma_{\max}}{S_{0}}\\ =(1-\frac{\sigma_{\max}}{S_{0}})\left[1-{\left(1-\frac{N}{N_{f}}\right)}^{u}\right] \end{array}$$

where *D_s_*(*N*) is the damage variable defined by the decay of the residual strength; the parameter *u* in the damage model is the same as that in the residual strength power function decay model, and the other parameters have the same meaning as Equation (9).

**Figure 18:**
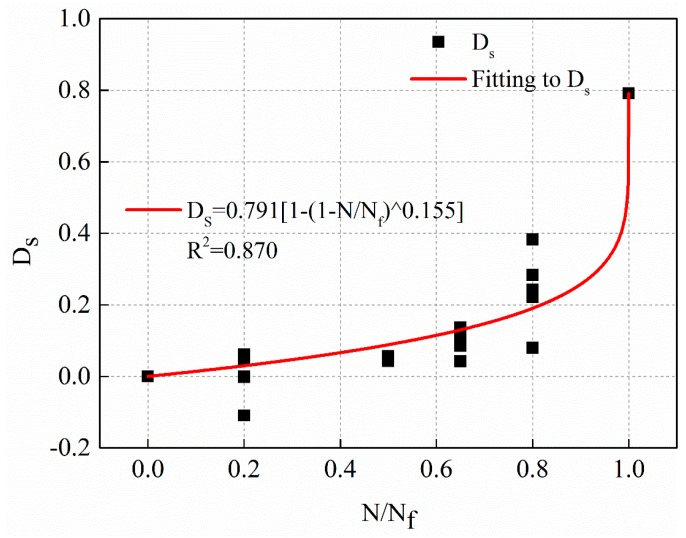
The fatigue damage evolution curve based on residual strength decay with stress levels of 1 MPa.

**Figure 19:**
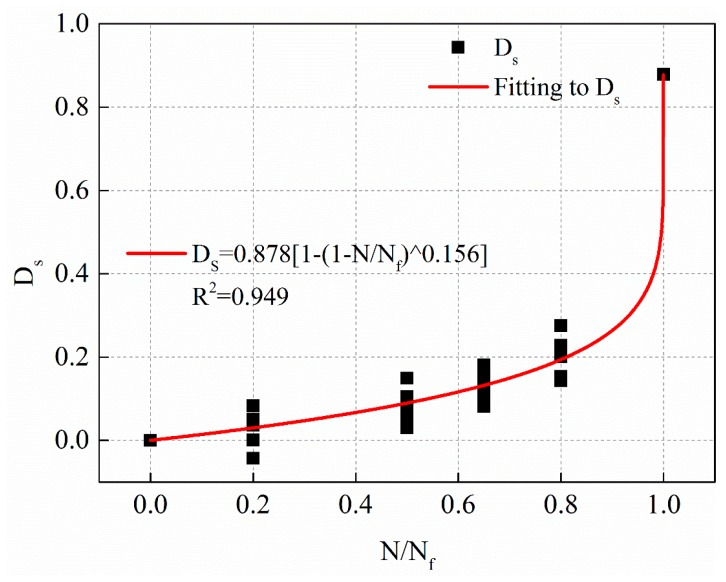
The fatigue damage evolution curve based on residual strength decay with stress levels of 0.5 MPa.

**Figure 20:**
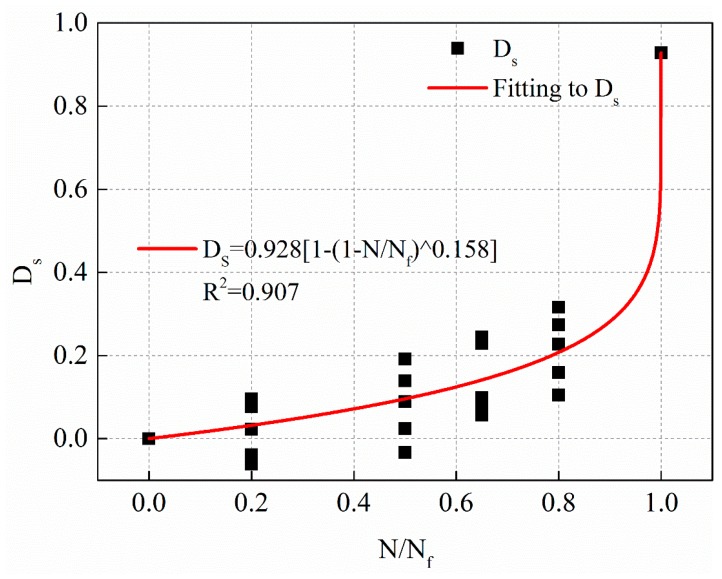
The fatigue damage evolution curve based on residual strength decay with stress levels of 0.25 MPa.

Nonlinear fatigue damage evolution curves under different stress levels are shown in Figure 18, Figure 19 and Figure 20, respectively.

The fitting equations of the nonlinear fatigue damage evolution curves in Figure 18, Figure 19 and Figure 20 are shown in Equations (21)–(23), respectively.

(21) $$1\;\mathrm{MPa}:\;D_{s}=0.791\left[1-{\left(1-\frac{N}{N_{f}}\right)}^{0.155}\right],R^{2}=0.870$$

(22) $$0.5\;\mathrm{MPa}:\;D_{s}=0.878\left[1-{\left(1-\frac{N}{N_{f}}\right)}^{0.156}\right],R^{2}=0.949$$

(23) $$0.25\;\mathrm{MPa}:\;D_{s}=0.928\left[1-{\left(1-\frac{N}{N_{f}}\right)}^{0.158}\right],R^{2}=0.907$$

The meaning of each parameter in the above Equations is the same as that of the Equation (20).

From the regression results, the regression parameter *u* values of different stress levels are very close. Value of *u* can be considered as a material constant considering the discreteness of the residual strength test data and the strength degradation is the most direct reason for structure failure. For the sake of simplification, the regression parameter *u* takes the average value of the three stress levels to be 0.156, the fatigue damage equation based on the residual strength decay law can be built as Equation (24) shows.

(24) $$D_{s}\left(N\right)=\left(1-\frac{\sigma_{\max}}{S_{0}}\right)\left[1-{\left(1-\frac{N}{N_{f}}\right)}^{0.156}\right]$$

**Figure 21:**
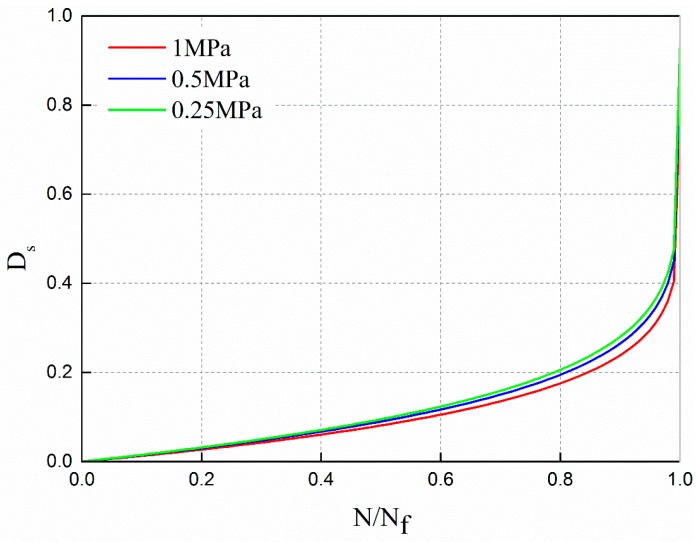
The damage evolution law based on residual strength decay under different stress levels.

In order to compare the fatigue damage evolution law based on residual strength decay under different stress levels, the curves are plotted in Figure 21 according to Equation (24).

It can be observed from Figure 21 that the fatigue damage evolution law under different stress levels is that at the beginning of the cycle ratio, the damage increases slowly. With the increase of the cycle ratio, the rate of damage increases gradually. Especially in the later stage of the cycle ratio, the growth rate has increased sharply. In the figure, the damage evolution curves are 1 MPa, 0.5 MPa and 0.25 MPa from bottom to top, respectively. The higher the stress level, the lower the damage evolution curve.

#### 4.3.3. Residual Strength-Dynamic Modulus Coupled Model 

**Figure 22:**
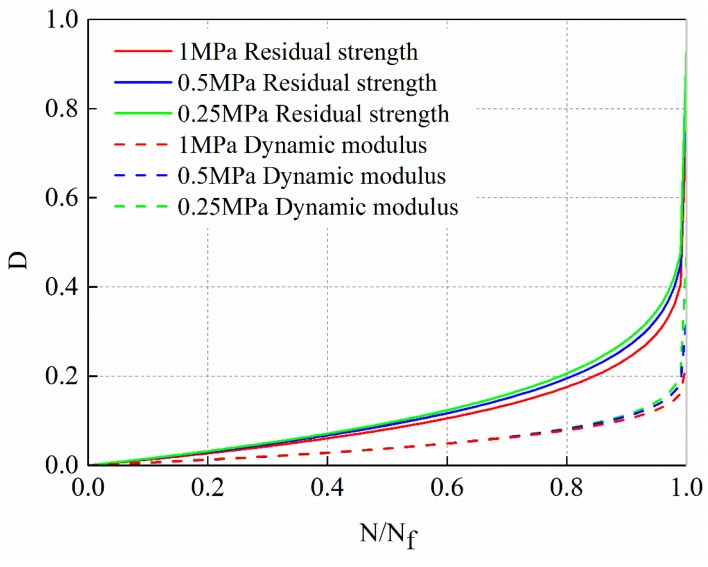
Comparison of damage evolution curves based on dynamic modulus decay and residual strength decay.

According to the dynamic modulus decay and residual strength decay, the damage variables of fine-grain asphalt mixture AC-13C in the same research object is defined and the fatigue damage evolution law is analyzed. The corresponding damage evolution model is established, as shown in Equations (18) and (24), respectively. The nonlinear damage evolution curves obtained from the two damage variables are plotted in Figure 22 for comparative studying.

From Figure 22, it can be observed that the fatigue damage evolution curve based on residual strength decay is significantly higher than based on dynamic modulus decay. It indicates that the damage based on residual strength decay is more extensive than based on dynamic modulus decay. In addition, the damage based on residual strength decay increases continuously from the initial stage. While the damage based on dynamic modulus decay increases slowly in the early and middle stages and increases sharply only near the failure stage. The damage defined by residual strength decay is more sensitive to evolution than that defined by dynamic modulus decay.

The reason for this difference of damage is a deterioration process of materials or structures caused by microscopic structural defects (such as micro-cracks and micro-voids). Damage affects the material properties through the changes in microstructure. The analysis of fatigue damage characteristics based on residual strength decay is essentially reflected by the change of strength characteristics in the process of fatigue. The strength characteristics are often determined by internal defects or damage of materials. It is a kind of material characteristic which is sensitive to internal structural defects or damage distribution. The damage variable based on residual strength decay can better describe the damage state of materials.

The fatigue damage defined by residual strength has natural physical criteria. The residual strength is consistent with the original definition of damage and is determined by internal defects or damage. The residual strength belongs to a kind of material property which is sensitive to internal structural defects or damage distribution. However, the residual strength is destructive and does not have continuity. One specimen can only get one experimental data point, and the comparability of residual strength between different specimens is poor. The dynamic modulus can be measured continuously in the process of fatigue test, which decreases monotonously with the accumulation of internal damage. The dynamic modulus can describe the damage state of asphalt mixture in the process of fatigue. Given this, combined with the advantages of residual strength and dynamic modulus in describing the damage, the damage variable *D_f_* defined by dynamic modulus is modified according to the standard of damage variable *D_s_* defined by residual strength.

It is assumed that the residual strength and dynamic modulus at the same time are determined by the same material damage state. The accumulated fatigue damage is an objective fact, and the residual strength and dynamic modulus have a certain relationship due to the same damage. The relationship between them could be built as Equation (25) shows.

(25) $$D_{s}=\left(1-\frac{S_{r}}{S_{0}}\right)=D_{f}{}^{\omega }={\left(1-\frac{E}{E_{0}}\right)}^{\omega }$$

where *ω* is the correlation coefficient of damage variable defined by residual strength decay and dynamic modulus decay; *D_s_* is the damage variable defined by residual strength decay; *S_r_* and *S*_0_ is residual strength and initial strength respectively; *D_f_* is the damage variable defined by dynamic modulus decay; *E* and *E*_0_ is dynamic modulus and initial dynamic modulus respectively.

**Figure 23:**
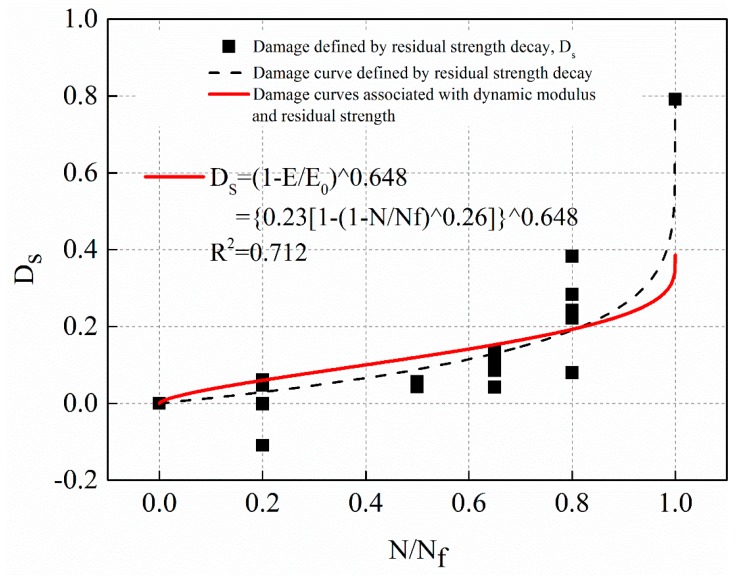
The damage evolution curve associated with dynamic modulus and residual strength with stress levels of 1 MPa.

**Figure 24:**
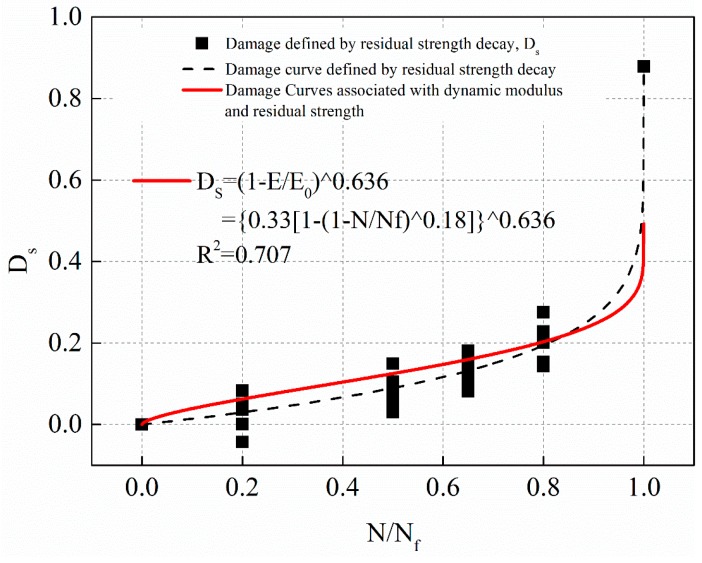
The damage evolution curve associated with dynamic modulus and residual strength with stress levels of 0.5 MPa.

**Figure 25:**
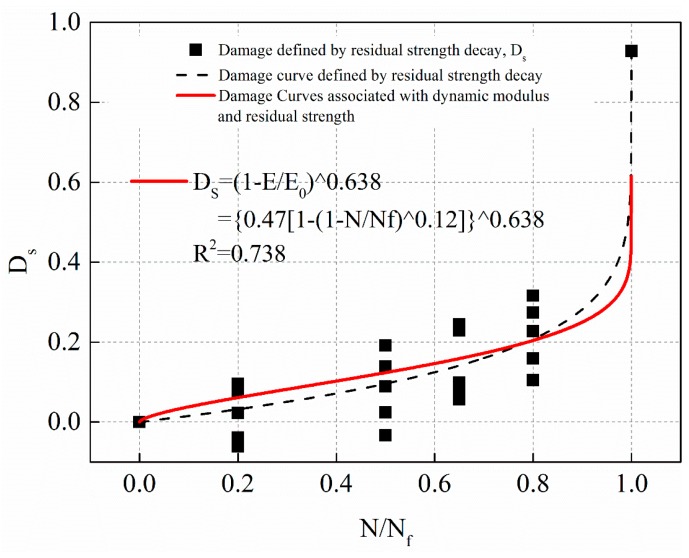
The damage evolution curve associated with dynamic modulus and residual strength with stress levels of 0.25 MPa.

Equation (18) was substituted into Equation (25). By fitting the damage of residual strength decay after different fatigue times, the correlation coefficient ω was determined. The fitting curves under stress levels 1 MPa, 0.5 MPa, and 0.25 MPa are shown in Figure 23, Figure 24 and Figure 25, respectively. The solid line in the figure was based on the damage fitting curve associated with dynamic modulus decay and residual strength decay. The dotted line is the fitting curve in which the damage is defined by residual strength decay alone in the study above. The fitting equations for the solid lines in Figure 23, Figure 24 and Figure 25 are shown in Equations (26)–(28), respectively.

(26) $$1\;\mathrm{MPa}:\;D_{s}={\left(1-\frac{E}{E_{0}}\right)}^{0.648},\;R^{2}=0.712$$

(27) $$0.5\;\mathrm{MPa}:\;D_{s}={\left(1-\frac{E}{E_{0}}\right)}^{0.636},\;R^{2}=0.707$$

(28) $$0.25\;\mathrm{MPa}:\;D_{s}={\left(1-\frac{E}{E_{0}}\right)}^{0.638},\;R^{2}=0.738$$

The fitting results of Figure 23, Figure 24 and Figure 25 show that the damage fitting curve based on the correlation between dynamic modulus decay and residual strength decay is high conformity with the damage data defined by residual strength decay alone. The damage fitting curve can well predict the damage evolution law of asphalt mixture. Under different stress level, the damage evolution law based on dynamic modulus decay or residual strength decay is different. However, the fitting value of damage correlation coefficient ω based on residual strength decay and dynamic modulus decay is very close. Considering the dispersion of the experimental data, it can be considered that the correlation coefficient ω is a material constant. In this paper, the average value of ω obtained at three stress levels is 0.641. Therefore, taking the damage variable defined by residual strength decay as the standard, the damage variable defined by dynamic modulus decay is modified as Equation (29) shows.

(29) $$D_{s}={\left(1-\frac{E}{E_{0}}\right)}^{0.641}$$

Based on the damage associated with dynamic modulus decay and residual strength decay, on the one hand, the damage defined by residual strength decay is consistent with the original definition of damage, and the accuracy of the fatigue damage model is improved. On the other hand, the damage defined by the dynamic modulus decay definition can be measured continuously.

## 5. Summary and Conclusions

In this paper, the residual strength decay and the dynamic modulus decay were used to evaluating the fatigue damage process of asphalt mixture AC-13C. The main conclusions are as follows:

(1) The main manifestation of the dynamic modulus decay and the residual strength decay is that the decay rate is relatively stable in the previous stage. When the cycle ratio is about 90%, the decay rate accelerates sharply until fatigue failure.
(2) The residual strength, dynamic modulus coupled model could better describes the fatigue damage evolution law of asphalt mixture. The value of parameter ω in the damage variable defined by the correlation residual strength-dynamic modulus concentrates in the [0.636, 0.648] interval. For current materials, ω could be treated as a material constant considering the dispersion of experimental data.
(3) A modified formula for calculating damage variables associated with residual strength and dynamic modulus was proposed.

Direct tensile fatigue test and residual strength test will be done with different kinds of asphalt mixtures in the future. Further study is required for asphalt mixtures with various coarse aggregates, or varying amounts of asphalt binder to verify the modified formula.

## Figures and Tables

**Table 1:** Test results of Styrene-Butadiene-Styrene (SBS) modified asphalt.

Test Projects		Test Results	Technical Requirements	Test Methods
Penetration (25 °C, 100 g, 5 s) (0.1 mm)		50.9	30–60	T 0604-2000
Penetration index PI		0.533 (r = 0.997)	≥0	T 0604-2000
Ductility (5 cm/min, 5 °C) (cm)		36.0	≥20	T 0605-1993
Softening point (Ring ball) (°C)		72.5	≥60	T 0606-2000
135 °C kinematic viscosity (Pa·s)		2.31	≤3	T 0620-2000
Solubility (%)		99.9	≥99	T 0607-1993
Flash point (°C)		267	≥230	T 0611-1993
Rolling Thin Film Oven Test (RTFOT) 163 °C, 85 min	Mass loss (%)	0.29	≤±1.0	T 0609-1993
	Residual penetration ratio (25 °C) (%)	78.0	≥65	T 0604-2000
	Residual ductility (5 °C) (cm)	24.3	≥15	T 0605-1993

**Table 2:** Properties of aggregate basalt.

Test Item	Test Results	Technical Requirements	Test Methods
Crushed stone value (%)	15.8	≤28	T 0316-2005
Los Angeles weared stone value (%)	19.2	≤30	T 0317-2005
Apparent relative density (g/cm^3^)	2.9	≥2.5	T 0321-2005
Water absorption (%)	1.3	≤2.0	T 0304-2005
Content of flat and elongated particles in coarse aggregate (%)	5	≤15	T 0312-2005
<0.075 mm particle content (Washing methods) (%)	0.1	≤1	T 0310-2000
Asphalt adhesion/grade	5	≥4	T 0616-1993
Firmness (%)	1.9	≤12	T 0314-2000
Content of soft stone (%)	2	≤5	T 0320-2000

**Table 3:** Properties of limestone mineral filler.

Test Item	Test Results	Technical Requirements	Test Methods
Apparent density (g/cm^3^)	2.702	≥2.5	T 0352-2000
Water content (%)	0.43	≤1	T 0353-2000
Appearance	No agglomeration	No agglomeration	-

**Table 4:** Summary of AC-13C mixture gradation.

Aggregate Size (mm)	Upper Gradation (% Passing)	Lower Gradation (% Passing)	AC-13C Gradation (% Passing)
16	100	100	100
13.2	100	90	95
9.5	85	68	74
4.75	68	38	48.5
2.36	50	24	34
1.18	38	15	23.5
0.6	28	10	15
0.3	20	7	11
0.15	15	5	8.5
0.075	8	4	6
Pan	0	0	0
Binder	SBS modified asphalt		
Aggregate	Basalt		
Mineral Filler	Limestone		

**Table 5:** Marshall Test results at optimal asphalt content.

Asphalt Aggregate Ratio (%)	Bulk Volume Relative Density (g·cm^−3^)	Volume of Air Voids VV (%)	Voids in Mineral Aggregate VMA (%)	Voids Filled With Asphalt VFA (%)	Marshall Stability (kN)	Flow Value (0.1 mm)
5.2	2.455	4.5	16.1	67.2	12.7	27.9
Specification Requirement [28]	-	3–6	>12.5	55–70	>8	15–40

**Table 6:** The test result of direct tensile.

Stress Level (MPa)	0.25	0.5	1
Average Fatigue Life (Times)	122,131	11,414	1719
CV%	54.6%	47.6%	21.8%

(CV is the coefficient of variation).

**Table 7:** The initial value of dynamic modulus (*E*_0_) of fatigue test.

Stress Level (MPa)	0.25	0.5	1
*E*_0_ (MPa)	5400	5460	5560
CV%	0.10	0.09	0.04

(CV is the coefficient of variation).

**Table 8:** The critical value of dynamic modulus (*E*_min_) of fatigue test.

Stress Level (MPa)	0.25	0.5	1
*E*_min_ (MPa)	2930	3435	4405
CV%	0.13	0.10	0.07

(CV is the coefficient of variation).

**Table 9:** Dynamic modulus decay parameter *m* under different stress levels.

Stress Level (MPa)	0.25	0.5	1
parameter (*m*)	0.121	0.179	0.260
CV (%)	0.124	0.095	0.071

**Table 10:** Design fatigue times at different stress levels.

The Cycle Ratio	Fatigue Times		
	1 MPa	0.5 MPa	0.25 MPa
${\bar{N}}_{f}$	1719	11,414	122,131
20% ${\bar{N}}_{f}$	344	2283	24,426
50% ${\bar{N}}_{f}$	860	5707	61,066
65% ${\bar{N}}_{f}$	1117	7419	79,385
80% ${\bar{N}}_{f}$	1375	9131	97,705
